# Qualitative Evidence Synthesis (QES) for Guidelines: Paper 1 – Using qualitative evidence synthesis to inform guideline scope and develop qualitative findings statements

**DOI:** 10.1186/s12961-019-0467-5

**Published:** 2019-08-08

**Authors:** Soo Downe, Kenneth W. Finlayson, Theresa A. Lawrie, Simon A. Lewin, Claire Glenton, Sarah Rosenbaum, María Barreix, Özge Tunçalp

**Affiliations:** 1 University of Central Lancashire, Preston, United Kingdom; 2 Evidence-based Medicine Consultancy Ltd, Bath, United Kingdom; 3 Norwegian Institute of Public Health, Oslo, Norway; 4 Health Systems Research Unit, South African Medical Research Council, Cape Town, South Africa; 5 Department of Reproductive Health and Research including UNDP/UNFPA/UNICEF/WHO/World Bank Special Programme of Research, Development and Research Training in Human Reproduction (HRP), World Health Organization, Geneva, Switzerland

**Keywords:** Evidence-to-decision, guideline development, GRADE, GRADE-CERQual, QES, qualitative review, qualitative evidence synthesis/syntheses, qualitative methods, WHO guidelines

## Abstract

**Background:**

WHO has recognised the need to ensure that guideline processes are transparent and evidence based, and that the resulting recommendations are relevant and applicable. Along with decision-making criteria that require findings from effectiveness reviews, WHO is increasingly using evidence derived from qualitative evidence syntheses (QES) to inform the values, acceptability, equity and feasibility implications of its recommendations. This is the first in a series of three papers examining the use of QES in developing clinical and health systems guidelines.

**Methods:**

WHO convened a group of methodologists involved in developing recent (2010–2018) guidelines that were informed by QES. Using a pragmatic and iterative approach that included feedback from WHO staff and other stakeholders, the group reflected on, discussed and identified key methods and research implications from designing QES and using the resulting findings in guideline development. Our aim in this paper is to (1) describe and discuss how the findings of QES can inform the scope of a guideline and (2) develop findings for key guideline decision-making criteria.

**Results:**

QES resulted in the addition of new outcomes that are directly relevant to service users, a stronger evidence base for decisions about how much effective interventions and related outcomes are valued by stakeholders in a range of contexts, and a more complete database of summary evidence for guideline panels to consider, linked to decisions about values, acceptability, feasibility and equity.

**Conclusions:**

Rigorously conducted QES can be a powerful means of improving the relevance of guidelines, and of ensuring that the concerns of stakeholders, at all levels of the healthcare system and from a wide range of settings, are taken into account at all stages of the process.

**Electronic supplementary material:**

The online version of this article (10.1186/s12961-019-0467-5) contains supplementary material, which is available to authorized users.

## Background

The external generalisability of guidelines compiled by WHO and other organisations has been a subject of debate [1–3]. One of the critiques has been the lack of consultation with guideline users and anyone affected by guideline recommendations about the scope and setting of priorities for guidelines. Another is that the lack of transparency, around how and which knowledge informs the recommendations, limits their external validity and transferability to a range of contexts, cultures and individuals [3].

WHO has recognised the need to improve its guideline methodology to ensure that guideline decision-making processes are transparent and evidence based, and that the resulting recommendations are relevant and applicable. Hence, the WHO Handbook for Guideline Development was produced. This stipulates that evidence of several criteria is required to inform a WHO guideline recommendation in addition to evidence of the effectiveness of an intervention [4]. These other criteria include values and preferences, acceptability, feasibility and equity implications. Qualitative evidence can help inform these criteria. More broadly, there is increasing interest in the use of qualitative evidence to inform decisions in a wide variety of sectors such as health and social care, prison care, and education. However, until recently, the decisions made by guideline panels about these criteria have been largely based on the expert opinion of guideline development groups at WHO and/or on evidence that they happen to know about or that has been collected ad hoc, rather than on a systematic review of relevant research [2].

A systematic review of qualitative studies, also known as a qualitative evidence synthesis (QES), is an approach for synthesising the findings from multiple primary qualitative studies. Findings from QES are generally more robust and useful than those from individual primary qualitative studies as they bring together evidence from multiple studies, thus providing richer data than a single study can. QES can also identify patterns in the data, explore similarities and differences across settings, lead to a new interpretive model or framework, and contribute broadly to a field of research.

In theory, evidence from QES can be used alongside effectiveness evidence [4] to inform all stages of developing a guideline, including identifying the relevant interventions and outcomes at the scoping stage, synthesising and evaluating evidence, formulating recommendations, and developing implementation considerations. QES reviews conducted at the scoping stage, before the guideline protocol is finalised, can identify broader concepts that can shape the overall scope of the guideline. Once the protocol is finalised, QES reviews designed to inform evidence-to-decision (EtD) frameworks are tailored to identify the acceptability, feasibility and/or equity of a specific intervention within the guideline, and/or to inform judgements about how much stakeholders might value the outcomes associated with the intervention.

The first WHO guideline that included QES was produced by the WHO Department of Reproductive Health and Research in 2012 [5]. Since then, this department has published at least six more guidelines that include QES findings [6–11], with two more in preparation (WHO: Guidance on communication interventions to inform and educate caregivers on routine childhood vaccination in the African region, under review; WHO: Recommendations on digital interventions for health systems strengthening, under review). In these guidelines, QES were used to inform the values and preferences, acceptability, feasibility, and/or equity criteria of the respective EtD frameworks. In two guidelines [7, 8], a priori conceptual QES were undertaken at the scoping stage to determine what outcomes were important to the relevant service users.

**Fig. 1:**
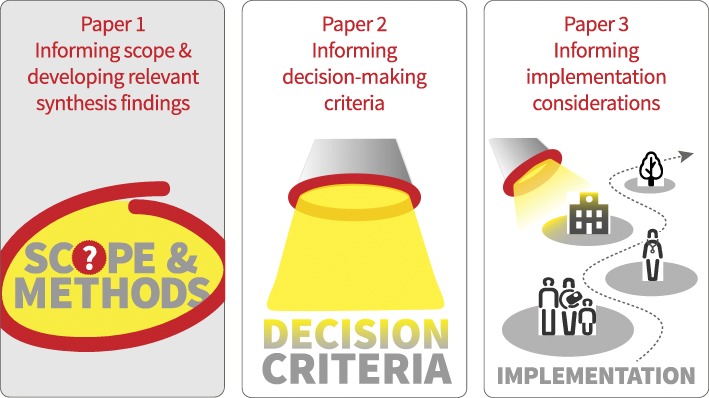
Qualitative evidence synthesis for guidelines: overview of this series of three papers

This paper is the first of a series of three on the use of QES to inform the development of clinical and health systems guidelines, drawing on experience from relevant WHO guidelines. The second and third papers deal with how findings can be used to populate key criteria for decision-making [12] and inform the implementation considerations of a guideline [13] (Fig. 1). Throughout the series, we explore the strengths and limitations of the described approaches, provide examples of what worked and what was less successful, and make suggestions for improvements. The series is aimed at guideline commissioners, members of guideline panels and guideline development researchers as well as qualitative review authors and primary qualitative researchers.

### The aim of this paper

We have aimed to describe and discuss methods for conducting a QES in the context of developing a guideline, so that QES findings can (1) inform the scope of a guideline and (2) be used to develop findings for key guideline decision-making criteria.

The paper starts with an overview of how studies are identified for inclusion in QES reviews, and then describes the different analytical approaches used for scoping reviews and developing findings.

## Methods

The experiences, guidance and data presented in this series of papers are the result of a range of processes that have evolved over a decade of engagement with qualitative research in the context of developing healthcare guidelines at WHO. To develop the ideas described in the series, we used a pragmatic and iterative approach that included the following steps:

1. WHO convened a core team of authors who had been involved in WHO guideline technical teams since 2010 and in developing QES to support these guidelines.
2. The core author team reflected on the guideline development processes in which it had been involved (see list below), focusing on the role of QES findings in these processes. We also received informal feedback on these processes from other WHO staff involved in guideline development and participants in several guideline training workshops at WHO. This reflection and feedback led us to identify the three key areas that became the focus of a paper each in the series – how QES can inform the guideline scope and develop findings for EtD frameworks; how to use findings from QES to populate the EtD frameworks; and how to use QES findings to develop implementation considerations and inform implementation guidance and processes.
3. The lead author for each paper then drafted an outline for their paper, and these outlines were discussed during a 4-day author workshop. In the workshop, authors discussed the most important factors in the use of qualitative evidence in this context to date and agreed on what worked and what could be improved in the future. The outlines were then developed into full papers, using an iterative process of sequential writing and discussion. We also identified relevant examples from the guidelines in which we had been involved. The core authors then reviewed the draft to clarify the ideas and processes described and add further examples, where needed.
4. We then circulated the draft papers to key stakeholders to obtain their feedback on the ideas and processes described. These stakeholders included members of WHO guideline panels, methodologists, guideline commissioners and implementation experts (see Acknowledgements).

We selected examples from the following WHO guidelines, in the compilation of which members of the core author team had been involved:

1. Optimising health worker roles for maternal and newborn health through task-shifting (2012) [5]
2. Expanding health worker roles to help improve access to safe abortion and post-abortion care (2015) [6]
3. WHO recommendations on antenatal care for a positive pregnancy experience (2016) [7]
4. WHO recommendations: intrapartum care for a positive childbirth experience (2018) [8]
5. Guidance on communication interventions to inform and educate caregivers on routine childhood vaccination in the African Region (under review)
6. WHO recommendations on digital interventions for health systems strengthening (under review)

All of these guidelines were health systems focused or had a health systems component, and all used the GRADE EtD frameworks [14]. The latter are documents with a common structure that includes a question, an assessment of the evidence that addresses the question, and a conclusion; this facilitates explicit and transparent decision-making [15] (see Additional file 1 for an example of an EtD framework template). Examples were selected to highlight how qualitative evidence was used in the guideline processes described and the strategies that we used to package this evidence for decision-making purposes.

**Fig. 2:**
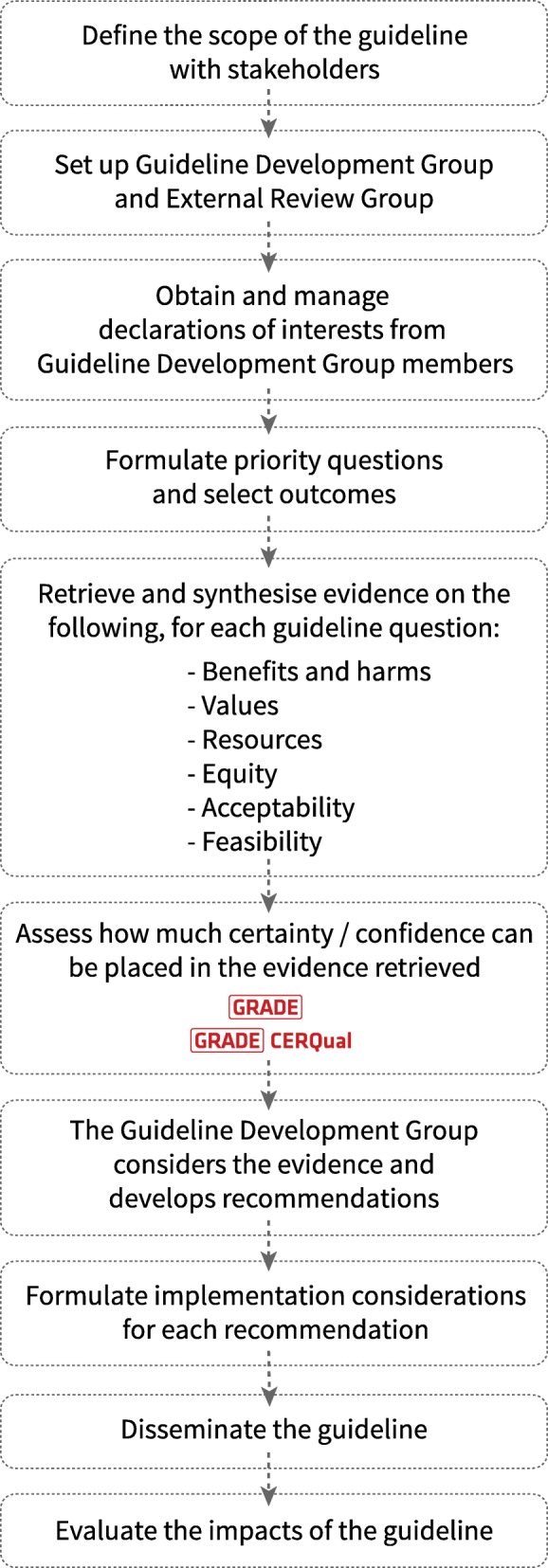
The WHO guideline development process

In some cases, we have made small changes to the wording of the examples so that they can stand alone from the guideline text or to emphasise the issue they are intended to highlight. We have noted in the text where we have adapted the examples from the guidelines listed above. The examples in this paper are derived mainly from the antenatal and intrapartum care guidelines [7, 8]. Figure 2 illustrates the WHO guideline development process.

## Results

We provide an overview here of how to conduct a QES in the context of guideline development, along with examples from QES-informed guidelines.

### Review protocol

The development of a protocol is important for framing the parameters of the review. Reviews undertaken for guidelines are often developed with commissioners, and sometimes also with stakeholders, to ensure relevance to policy. This means that the period of protocol development may be slower than for researcher-driven reviews. Once a protocol has been agreed upon, it is good practice to ensure it is publicly available before the review commences. This can be done by registration with a relevant organisation, body or agency (e.g. EPOC [Effective Practice and Organisation of Care] or PROSPERO [International Prospective Register of Systematic Reviews]), or through publication in a journal. As in other qualitative systematic reviews, the protocol should include the objective of the review, criteria for including studies (types of studies, participants, settings, interventions and phenomena of interest), the search strategy, data collection and analysis, and a reflexivity statement.

### Reflexivity statement

The reflexivity statement expresses the a priori views, values and beliefs of the review authors about the subject of interest. It is intended to provide some transparency and give readers an insight into the lens through which the authors have viewed their data. For example, the reflexivity statement in the QES conducted to populate the antenatal care (ANC) guideline EtD frameworks states:

“*In keeping with quality standards for rigour in qualitative research, the review authors considered their views and opinions on antenatal care as possible influences on the decisions made in the design and conduct of the study, and, in turn, on how the emerging results of the study influenced those views and opinions. All review authors believed at the outset, that contact with formal and informal caregivers throughout pregnancy was valuable, but that formal antenatal care provision is generally over-focused on clinical procedures and the assessment of risk/ill-health, with too little focus on psychosocial aspects of pregnancy. We therefore used refutational analytic techniques (‘disconfirming analyses’) to minimize the risk that these presuppositions would skew the analysis and the interpretation of the findings*” [16].

### Search methods

Ideally, an initial scoping search should be conducted prior to the framing of the guideline parameters to identify potential concepts, e.g. values and associated outcomes that may be important to the population under investigation. Where this has been done, the findings from the scoping review may guide the subsequent QES search criteria, e.g. the ANC guideline scoping review highlighted a number of (non-clinical) aspects of ANC that were particularly important to women (care, support and information) but that had not been initially highlighted by the guideline development group. We therefore included search criteria such as ‘support’ in the search strategy for the subsequent QES designed to generate specific findings. For both searches, the search strategies should be transparent, with clear parameters and filters where appropriate.

### Preparing an effective search strategy

In preparing the search strategy, consideration should also be given to the following characteristics:

1. Databases – A selection of relevant databases should be identified by the review group, incorporating those that index studies from specific settings, where appropriate. In the antenatal and intrapartum care reviews, we wanted to maximise global reach, so we utilised LATINDEX for studies from South America, and AJOL (African Journals Online) to pick up studies from Africa as well as more commonly used databases such as MEDLINE, CINAHL and PsycINFO.
2. Date range – If a range of dates is relevant to the review objective(s) this should be identified by the reviewers. For example, in the ANC QES, we used 2001 as a start date as this was when the existing WHO ANC programme was introduced.
3. Types of publications – Reviewers should look for studies utilising a qualitative research design (e.g. ethnography or phenomenology) or qualitative methods for data collection (e.g. focus group interviews, individual interviews, observation, diaries and oral histories), and which use qualitative methods for data analysis (e.g. thematic analysis, framework approach, grounded theory and thematic network analysis). Mixed-methods designs may also be relevant where findings are from the qualitative component. Reviewers should be clear about the documents they are looking for and decisions will need to be taken about the inclusion of grey literature, unpublished studies, commentaries, reports, etc.
4. Language – Where appropriate, we would encourage reviewers to include studies published in languages other than English, particularly if the review has a global context. However, the ability of reviewers to translate relevant studies needs to be considered, as additional resources may be required here. In the reviews performed for the antenatal and intrapartum care guidelines, we used ‘Google Translate’ (https://translate.google.com) to perform an initial translation and, if the study appeared to be relevant, we identified sources within WHO to provide a more formal translation.

### Study selection

The processes used to identify studies for QES in the context of guidelines are similar to those of other systematic reviews. Studies should be screened and selected based on the predetermined inclusion and exclusion criteria highlighted in the protocol. Careful consideration of these criteria and their relevance to the study objectives will help to focus the scope of the review and limit the number of papers selected to a manageable amount. Reviewers should make every effort to ensure that the search strategy optimises the opportunity to locate the maximum number of studies from the full range of contexts and participants for which/whom the guideline is intended to apply. However, unlike the techniques used to identify quantitative studies for systematic reviews or meta-analyses, it is not essential to identify and include every available relevant study. The purpose of QES is interpretive rather than predictive. Important, transferable concepts (or themes) are unlikely to change substantially in subsequent studies once they are consistently found in a body of papers from a wide range of participants and contexts. The number of studies included in any specific QES will therefore depend on the variety of concepts identified, the range of sociocultural contexts of interest to the guideline, and the degree of agreement between studies on the emerging concepts and themes.

For the QES conducted to inform the antenatal and intrapartum care guidelines, respectively [17, 18], study selection was done by the review lead authors, one of whom was an expert in the field. As these guidelines were about healthy women receiving routine care, we excluded studies that fell outside of these parameters such as those including only women with specific pregnancy complications. Where there were doubts about specific studies, agreement on inclusion/exclusion was reached by consensus or adjudication by a third reviewer [15, 16].

### Assessment of quality

The included studies should be subjected to a formal quality appraisal using one of the recognised appraisal systems for qualitative research. There is as yet no standardised tool for this process, but a modified version of CASP (Critical Appraisal Skills Programme) was used for the OptimizeMNH guideline QES [17, 19], whilst an amalgamated tool, incorporating the characteristics of several qualitative appraisal tools, was used for the antenatal and intrapartum care QES [20, 21]. This latter instrument rates studies against 11 criteria, and then allocates a score of A–D to each study, based on the extent to which it demonstrates credibility, transferability, dependability and conformability. Studies scoring D, which have “[s]*ignificant flaws that are very likely to affect the credibility, transferability, dependability and/or confirmability of the study*”, are excluded on the grounds of methodological quality.

### Sampling

In the examples discussed in this paper, the number of included studies ranged from 35 to 53 [17, 19]. When there is a large number of included studies or a disproportionate number of studies from a particular country or context, reviewers may wish to select a sample. A ‘large number’ is difficult to quantify and will, to a certain extent, depend on the emerging themes and concepts as well as the resources available and the timeframe required to complete the review. Reviewers should seek to ensure that no one sampling system affects the overall quality of the review by introducing reviewer bias. In the OptimizeMNH reviews, the authors “*utilized purposive sampling in order to arrive at a group of studies that provided geographical coverage*” [17, 19]. By achieving this coverage, the authors hoped to ensure a greater variation in contexts and thereby greater conceptual diversity. There are a number of sampling methods as well as a variety of approaches, and reviewers should be aware of the different techniques before deciding which to use [17, 18, 22, 23].

### Demonstrating rigor in study selection

For both types of QES reviews, the standard PRISMA flow diagram should be presented to demonstrate the decisions that led to the final study inclusion (see Additional file 1 for a PRISMA flow diagram from the intrapartum care review).

### QES at the guideline scoping stage

Scoping is the first stage of guideline development. It entails agreement with stakeholders about which interventions and which outcomes are most significant for the review. The process is informed by what evidence already exists (usually in the form of systematic reviews of existing randomised trials of effectiveness) as well as by what interventions are most likely to influence the health and well-being of the target population. Decisions about critical and important outcomes that will underpin the recommendations in the guideline must take into account what outcomes have been measured in the relevant systematic reviews of effectiveness as well as those that are important in practice for the guideline stakeholders, including clinical or policy experts, health professionals and service-users.

Although stakeholders with experience in practice or in health system development and implementation provide important expertise regarding outcomes that matter at a broad population level, patients and service-user groups have been concerned about a lack of attention to outcomes that matter to them directly. For example, for some years, pregnant and child-bearing women have levelled the critique that, whilst most women and babies are healthy, the standard outcome measures for maternity care interventions tend to be focused on pathological outcomes. In recognition of the importance of going beyond just surviving the consequences of pregnancy and childbirth, the United Nations ‘Survive, Thrive, Transform’ agenda has shifted the perspective of maternity services from an emphasis on only reducing mortality and morbidity to one that is about both survival and well-being [24]. The scoping process for the recent WHO antenatal and intrapartum care guidelines recognised the need to expand expert professional consideration of relevant outcomes to include the views and experiences of child-bearing women [7, 8]. Based on this change of focus, two scoping reviews of what matters to women in relation to pregnancy and childbirth, respectively, were conducted to inform these guidelines [18, 25].

### Methodology of the scoping reviews for WHO antenatal and intrapartum care guidelines

The intention of these scoping reviews was to find out what mattered to women socially, psychologically, emotionally and physically, independent (as far as possible) of their experience of local maternity services. We used established methods for the synthesis of qualitative data [26]. These are extensively described in the primary outputs of the scoping reviews [18, 25] and in methods papers relating to qualitative synthesis [16, 26, 27]. A summary of the qualitative synthesis methods used is given in Box 1; we used ‘meta-ethnography’ as the synthesis technique [16].

### Findings from the scoping reviews

The themes emerging from the data in the included scoping reviews led to ‘line of argument’ syntheses (Box 2). A robust line of argument is more than the sum of the parts of the review. It has high theoretical transferability beyond the specific included studies, and so it is likely to be applicable in a wider range of settings and circumstances. For both the antenatal and intrapartum care guidelines [18, 25], the lines of argument derived from the scoping reviews were used to inform and direct the philosophical framing of the guideline recommendations. The findings illustrated that what matters to women around the world in relation to both pregnancy and childbirth is both safety (physical, clinical, psychological and emotional) and a positive experience. These components were then summarised into a single composite outcome for each review, termed ‘positive pregnancy experience’ and ‘positive childbirth experience’, respectively. Box 2 provides more detail on the components of these outcomes. It shows that the positive experience concept captures factors that are part of the standard outcomes dataset for maternity care effectiveness reviews, i.e., mortality and morbidity. However, they also encompass factors that map directly into the ‘Survive, Thrive, Transform’ agenda such as psychosocial and emotional outcomes in both the short and longer term.

### The value of the novel ‘positive experience’ outcomes

The overall notion of a positive experience (of both pregnancy and childbirth) has never been part of standard outcomes assessment for studies of maternity care interventions. The nearest equivalent is ‘satisfaction’. However, satisfaction is known to be a poor measure of healthcare experience and it does not encompass the multiple dimensions of positive well-being identified in Box 2. In recognition of the importance of these findings, both the antenatal and intrapartum care guideline panels adopted the new positive experience outcome for their effectiveness reviews. Since it did not exist before the guideline process, there were no pretested measurement tools associated with it, so none of the effectiveness trials included in the review captured it directly (if at all). However, it was included to ensure that any proxy measures (including satisfaction) used in the eligible trials were identified and flagged, and as a driver for the development of positive experience outcome measures for use in future trials and reviews.

The notion of a positive experience was also adopted by the guideline development groups as the overarching guideline concept. It even influenced the guideline title ‘WHO Recommendations on Antenatal Care for a Positive Pregnancy Experience’ [7]. For the ANC guideline, further framework analysis conducted during the scoping QES informed the WHO vision for ANC and the overarching aim of the new WHO ANC model, which is “*to provide pregnant women with respectful, individualised, person-centred care at every contact, with implementation of effective clinical practices (interventions and tests), and provision of relevant and timely information, and psychosocial and emotional support, by practitioners with good clinical and interpersonal skills within a well-functioning health system*” [7]*.*

The lines of argument from these scoping reviews also highlighted certain stakeholder beliefs and values that had been overlooked by the respective guideline development groups. For example, in the scoping review for the intrapartum care guideline [18] women’s concerns about pain and pain-relief during labour were a significant finding and one which had been excluded from the initial guideline development discussions. Questions relating to pain-relief options were subsequently added in the guideline development process and incorporated into the final guideline [8]. Similarly, guideline questions were added to the ANC guideline development process on interventions for physiological symptoms, midwife-led continuity of care and group ANC, following the QES findings on ‘what matters to women’ about ANC [25].

### QES to develop summary of findings statements for decision-making (EtD Frameworks)

Whereas a priori scoping reviews are broad and conceptual, qualitative reviews to develop findings for key EtD framework criteria within the guideline protocol are directed by the types of interventions that are being examined. Therefore, the approach to synthesising data from included studies for this purpose should be more focused than for scoping reviews.

### Data analysis

Once the included studies have been identified and appraised for methodological limitations, the analytic phase can begin. The main purpose of an EtD-orientated QES is to generate a series of findings from the included data, which are directly focused on interventions addressed in the guideline, assessed for confidence and tailored towards acceptability, feasibility and equity, and the values that stakeholders attribute to the outcomes associated with the intervention. The findings are then added to the guideline EtD frameworks, prior to guideline panel consideration, as discussed in the second paper in this series.

For the antenatal and intrapartum care QES, as for the scoping QES methods, we firstly identified an index paper and built the themes with the data from subsequent papers (Box 1). Unlike for the scoping QES, we then unpacked the detail of the final themes into short statements (findings). These were then subject to appraisal [28] to determine the degree of confidence placed on each finding (Table 1). For the appraisal process, we used a relatively new technique, Confidence in the Evidence from Reviews of Qualitative research (CERQual), part of the GRADE tools for appraising findings from systematic reviews. The CERQual tool includes four appraisal components (methodological limitations, relevance, coherence and adequacy) and each finding is assessed against these criteria before being given an overall grade (high, moderate, low or very low confidence). For more information on the [ of CERQual, readers are referred to a recent series on the topic [29–35].

**Table 1:** Themes, short-form Summary of Findings, and CERQual ratings for the WHO antenatal care guideline. The Summary of Findings that emerged from both women and provider data on their views and experiences of antenatal care

Theme	Short-form Summary of Findings by CERQual rating		
	High confidence	Moderate confidence	Low/very low confidence
Sociocultural context	Pregnancy seen as a normal event	Cooperation with influential community members^a^	Gender of healthcare provider
Service philosophy, design and provision	Indirect cost of services	Poor infrastructure Long waiting times Staff training^a^	Staff corruption^a^
What matters to women and staff	Authentic and kind staff Antenatal care as a source of knowledge and information	Continuity of care	Attraction of specific components of antenatal care^a^

^a^ From providers only

### Tailoring QES findings statements for EtD frameworks

Once the review findings for a QES have been generated, reviewers should start drafting short statements that describe the findings data. The statements associated with each finding need to be framed with end-users and key stakeholders in mind, and the review team should consider what these potential users would want to know [15].

Each finding statement should be clear and concise and accurately capture the meaning of the underlying data that contribute to it. Each one should include an assessment of confidence in the contributing evidence. A finding statement should be developed iteratively so that key concepts can be clarified and explored, but it should be no more than a few sentences in length. For example, in the short form of findings represented in Table 1, women and service providers consistently reported on a variety of issues relating to the influence of others, indirect cost of services, time spent with the professional or service user (depending on the perspective), flexibility of appointments, and continuity of care.

The full finding statement for ‘Indirect cost of services’, for example, was “*In the vast majority of countries ANC is provided free of charge but in many contexts the indirect costs associated with transport to and from the clinic, the purchase of additional medicines and the potential loss of income associated with clinic attendance all act as a barrier to engagement with ANC*” (high confidence in the evidence).

Readers should bear in mind that there may be contradictory review findings on the same or similar issues. Reviewers need to strike a balance between splitting issues emerging from the synthesis into multiple review findings, resulting in findings that are no longer useful to end users and do not fully represent the phenomenon of interest, and generating a smaller number of broad findings that oversimplify or fail to adequately capture variations across different contexts. For example, in the QES on experiences of intrapartum care, women talked about the importance of the birthing environment. Sometimes, these views were expressed positively (e.g. health facilities were described as clean and safe) and, in other instances, they were expressed negatively (labour wards were deemed to be unhygienic, poorly maintained and overcrowded). These opposing views could be reflected as two separate statements but, in this case, following discussions between the review authors, the finding statement was captured under a broad heading that encapsulated both sets of experiences, i.e. the need for a safe and secure environment: “*Women highlighted the importance of having a safe, clean and relatively private space to give birth and emphasized the importance of having medical equipment on hand should the need arise. Where these criteria weren’t in evidence, women felt unnerved by the lack of space, poor hygiene, state of disrepair and the loss of dignity associated with giving birth in overcrowded delivery rooms*.” The decision to retain the integrity of opposing views as separate and distinct may depend on prior knowledge of the phenomena and/or the relative importance in different contexts or amongst different population groups. For example, in the QES on women’s experiences of ANC, some women reported that providers could be rude, disrespectful and occasionally abusive, while others highlighted qualities of care, compassion and kindness amongst providers. In theory, these divergent views could be merged under a heading like ‘staff attitude’ but, on closer inspection, it became clear that the women reporting rude and abusive behaviour were generally located in low-income settings, whilst those discussing kindness were largely resident in high-income countries. We therefore took the decision to express these findings separately as ‘Rude and abusive staff’ and ‘Authentic and kind staff’.

**Fig. 3:**
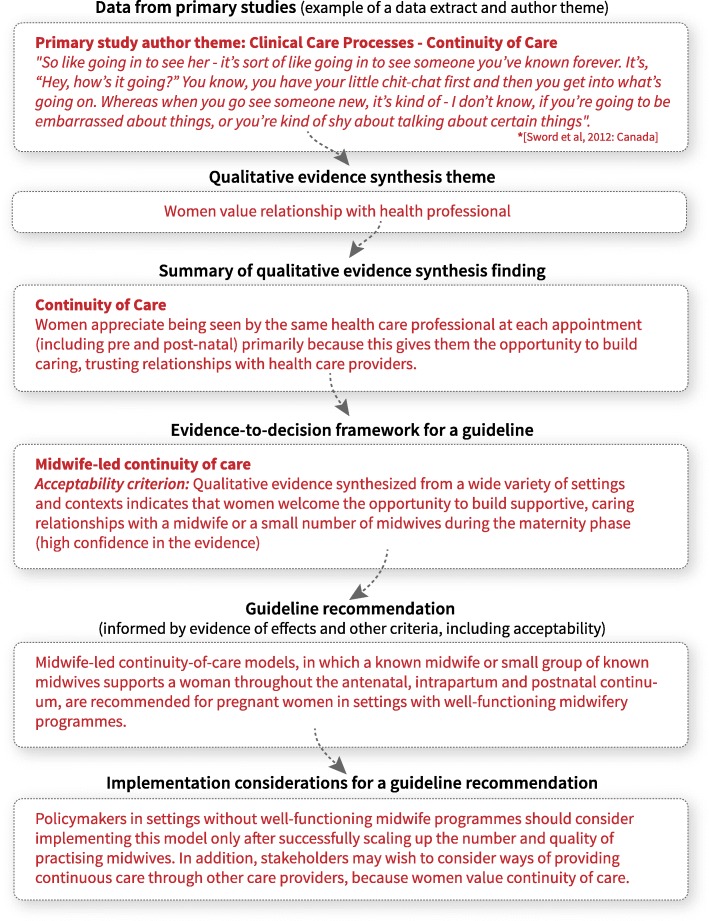
The journey of qualitative data – how data from primary qualitative studies informs guideline recommendations

Figure 3 provides an example that illustrates the journey of an individual quote from a primary paper through the guideline development process. Approaches for populating GRADE EtD frameworks with qualitative findings are covered in the next paper in this series [12].

### Demonstrating rigor in study analysis (for both types of QES)

As for all systematic reviews, the characteristics and quality assessment of the included studies should be presented in the review, along with a summary of the reasons for excluding studies. A table listing the review themes and/or a Summary of Findings should be included, which lists the codes for the included studies that contributed to each theme or finding. For each finding included in the Summary of Findings, the CERQual rating should also be listed alongside, with reasons for downgrading if this has occurred. A finding may be downgraded if it fails to meet any of the four appraisal components (methodological limitations, relevance, coherence and adequacy) inherent in the CERQual tool. This can also be done for thematic findings. Examples of this approach can be found in the QES papers on women and health provider experiences of antenatal and intrapartum care [16, 27].

## Discussion

In this paper, we have tried to describe and discuss the methods for conducting a QES in the context of developing a guideline and have explored how QES findings can inform the scope of a guideline and be used to develop findings for key guideline decision-making criteria.

The limitations of this approach to guideline development include the difficulty of determining at what stage in the guideline development process the review should be undertaken, and the additional resources required to conduct the review rigorously. In our experience, the process of undertaking qualitative reviews (particularly scoping reviews) identified factors that were important to stakeholders but that had not been considered in the prior guideline group agreements about which effectiveness reviews to include. This suggests that undertaking the qualitative reviews earlier might have improved the scope of the final guidelines. For other guidelines, it became clear that some sub-questions could have benefited from more focused qualitative reviews earlier in the process. For example, for the ANC guideline, separate QES reviews could have been conducted of women’s experiences of nutritional, maternal and fetal assessment, preventive measures, and physiological and health systems interventions. This lesson was learned for the intrapartum care QES, in which ‘mini-reviews’ of the qualitative evidence in specific areas (such as episiotomy) were undertaken as the guideline development process progressed. The need for these kinds of supplementary reviews is not always predictable at the commissioning stage, and therefore also has resource implications.

The benefits of committing additional resources to the examination of qualitative evidence include the potential for reframing guidelines to fit with real-world concerns and issues for key stakeholders. It also provides an opportunity to maximise the impact of the resulting recommendations in practice for those funding, providing and using services. In the case of the guidelines that underpin this series, the inclusion of qualitative data encouraged the guideline panels to pay particular attention to the experiences of women, providers and policy-makers at all stages of the process, from the overall values that framed the guidelines, through to implementation considerations and design of implementation tools and processes. This observation aligns closely with the hypothesised benefits of both realist and implementation science, in which the interest is to understand not just what works, but how it works (best), in what contexts, and for which individual(s) and groups [36].

Beyond these practical considerations, in our experience with two of the included guidelines [7, 8], the inclusion of an a priori scoping review changed the focus of the guideline dramatically. In both cases, this was a shift from an almost exclusive intention to reduce pathology, towards a need for maternity care to also catalyse positive maternal, newborn and family experiences. This legitimised the inclusion of effectiveness reviews on topics such as respectful care, companionship in labour and continuity of midwife care, alongside interventions such as pain-relief options.

Future methodological research in this area could include an examination of the degree to which qualitative review findings shape the scope of guidelines, inform the interventions to be included and impact on the information given to panel members, how this information is received and translated into recommendations, and the impact of implementation in practice. Cost-effectiveness considerations should also be examined, particularly in relation to the levels of skills and experience needed in qualitative review teams, and the degree to which qualitative reviews can be iteratively commissioned as the need for new information emerges from the work of specific guideline development groups.

## Conclusions

A guideline should aim to be as relevant as possible to those who are affected by the recommendations, including health service users, care providers, funders and service managers. The scope of the guideline should, therefore, reflect the needs of stakeholders. Many guideline development groups include representatives of the groups most affected by the recommendations such as people living with particular health or social issues. Whilst the views of these representatives are important, they are unlikely to represent all groups affected, particularly for global guidelines such as those produced by WHO. Members of guideline development groups, such as service providers and academics, are not always the best judges of what matters to patients or other service users. QES findings are, therefore, a good source of information about what matters to different groups of people who may be affected by a guideline.

In this paper, we have described how QES can influence the scope of guideline development and inform the criteria for decision-making in the context of healthcare guidelines. We have shown the importance of initial conceptual reviews at the scoping stage for determining meaningful outcomes and prioritising which interventions are to be included. As part of our practical guide for those who will contribute QES reviews to future guidelines, we have provided examples of good practice in undertaking and interpreting QES reviews as well as insights into the potential pitfalls. Our experience has shown that rigorously conducted qualitative reviews can be a powerful means of improving the relevance of guidelines and ensuring that the concerns of stakeholders, at all levels of the healthcare system and from a wide range of settings, are taken into account at all stages of the process.

Box 1. Summary of qualitative synthesis methods used for the QES at the guideline scoping stage Step 1. The included papers were examined, and an index paper was selected that best reflected the focus of the review.

Step 2. The themes and findings identified by the authors of this paper were entered onto a spreadsheet, to develop an initial thematic framework.

Step 3. The findings of all the remaining papers were then mapped into this framework, which continued to develop as the data from each paper were added. This process included looking for what was similar between papers (‘reciprocal analysis’) and what contradicted (‘disconfirms’) the emerging findings (‘refutational analysis’). For the refutational process, as each paper was added to the analysis, we consciously looked for data that could disconfirm our emerging themes or our prior beliefs related to the topic of the review. If any disconfirming data were found, the themes were amended, so that they continued to capture all the data from the papers we had already analysed as well as taking account of the new insights.

Step 4. All the themes were translated (or synthesised) into a ‘line of argument synthesis’, based on theoretical concepts that explain the data at a conceptual level.

Box 2. Line of argument syntheses from two scoping QES to inform WHO guidelines 1. What matters to women: a scoping review to identify the processes and outcomes of ANC provision that are important to healthy pregnant women [28]

“*Women want and need a positive pregnancy experience, including four subthemes: maintaining physical and sociocultural normality; maintaining a healthy pregnancy for mother and baby (including preventing and treating risks, illness and death); effective transition to positive labour and birth; and achieving positive motherhood (including maternal self-esteem, competence, autonomy).*”

What matters to women during childbirth: a systematic qualitative review.

2. “*For most childbearing women across the world, there is inherent value in being able to use one’s own physical and psychosocial capacities to labour, and to give birth to a healthy baby, even when the process is unpredictable and painful. Beliefs about what matters to women are influenced by familial experiences, and local cultural norms and values. The capacity for women to enact what matters to them is affected by anticipated or actual encounters with maternity care staff and services, including the use of desired, required, and/ or feared childbirth interventions.*”

## Additional file

Additional file 1: PRISMA Flow Diagram – What matters to women during childbirth. (PDF 215 kb)

## Data Availability

Not applicable.

## Abbreviations

ANC: antenatal care
CERQual: Confidence in the Evidence from Reviews of Qualitative research
EtD: evidence-to-decision
GRADE: Grading of Recommendations, Assessment, Development and Evaluation
QES: qualitative evidence synthesis/syntheses
