# Supplementary material for: Qualitative Evidence Synthesis (QES) for Guidelines: Paper 1 – Using qualitative evidence synthesis to inform guideline scope and develop qualitative findings statements
Source: Health Res Policy Syst. 2019 Aug 8;17:76. doi: 10.1186/s12961-019-0467-5 (PMC6686511; doi:10.1186/s12961-019-0467-5)
Supplement: Supplementary file 1 — PRISMA Flow Diagram – What matters to women during childbirth. (PDF 215 kb) [file 12961_2019_467_MOESM1_ESM.pdf]

PRISMA Flow Diagram – What matters to women during childbirth.

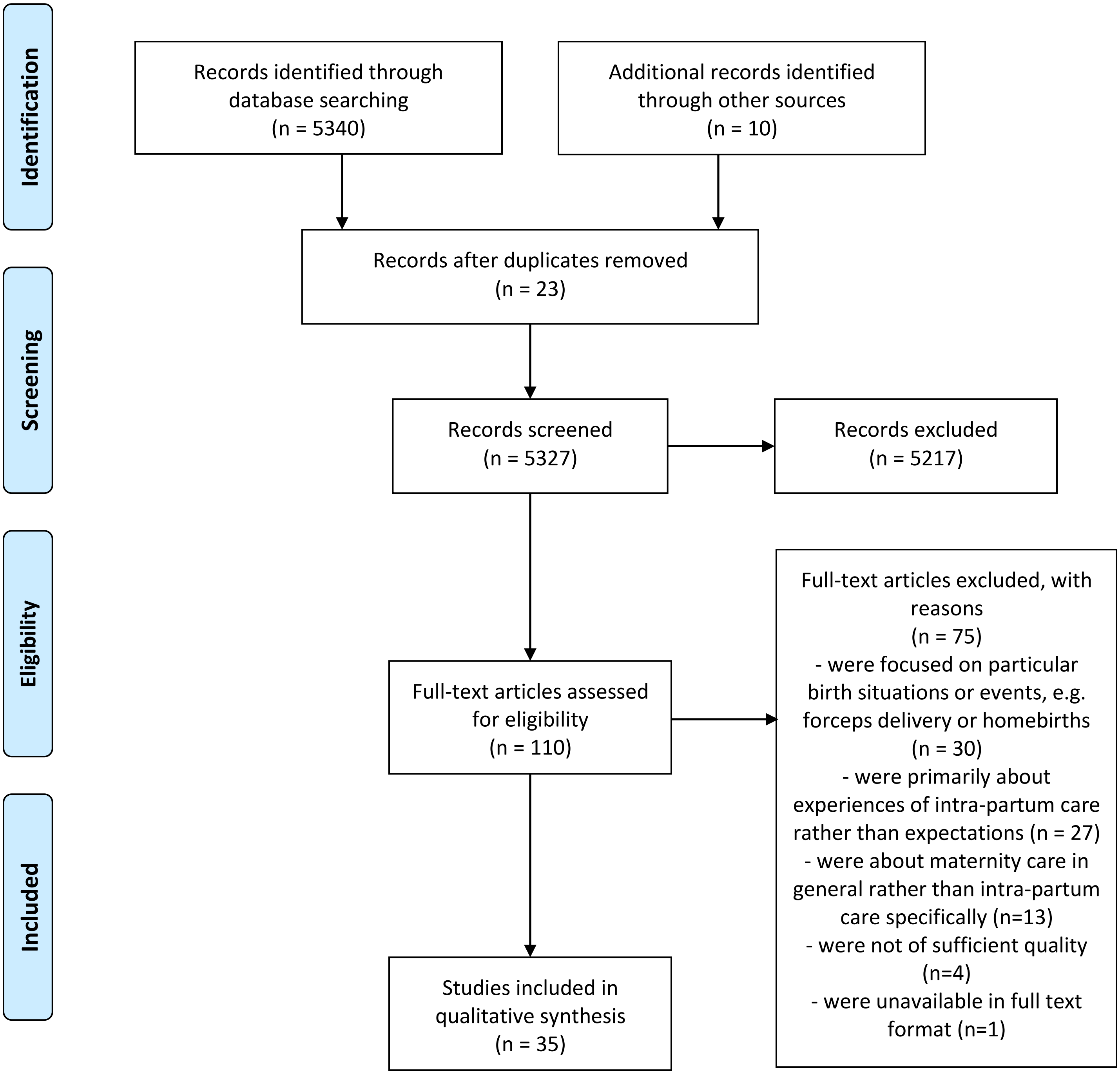

From: Moher D, Liberati A, Tetzlaff J, Altman DG, The PRISMA Group (2009). *P*referred *R*eporting *I*tems for *S*ystematic Reviews and *M*eta-*A*nalyses: The PRISMA Statement. PLoS Med 6(6): e1000097. doi:10.1371/journal.pmed1000097

For more information, visit www.prisma-statement.org.
